# Analyzing the impact of pharmacogenomics-guided nonsteroidal anti-inflammatory drug alerts in clinical practice

**DOI:** 10.1093/jamiaopen/ooaf112

**Published:** 2025-10-03

**Authors:** Amanda Massmann, Natasha J Petry, Max Weaver, Halle Brady, Roxana A Lupu

**Affiliations:** Sanford Imagenetics, Sanford Health, Sioux Falls, SD 57105, United States; Department of Internal Medicine, University of South Dakota School of Medicine, Vermillion, SD 57069, United States; Sanford Imagenetics, Sanford Health, Sioux Falls, SD 57105, United States; Department of Pharmacy Practice, North Dakota State University, Fargo, ND 58102, United States; Sanford Imagenetics, Sanford Health, Sioux Falls, SD 57105, United States; Sanford Imagenetics, Sanford Health, Sioux Falls, SD 57105, United States; Department of Internal Medicine, University of South Dakota School of Medicine, Vermillion, SD 57069, United States; Sanford USD Medical Center, Sanford Health, Sioux Falls, SD 57105, United States

**Keywords:** anti-inflammatory agents, non-steroidal, Cytochrome P-450 CYP2C9, pharmacogenetics, decision support systems, clinical, electronic health records

## Abstract

**Objectives:**

This study evaluates response rates of pharmacogenomics (PGx) nonsteroidal anti-inflammatory drugs (NSAIDs) clinical decision support (CDS) alerts at Sanford Health from May 2020 to December 2024.

**Materials and Methods:**

A retrospective analysis was conducted on PGx NSAIDs interruptive alerts. Response options were classified into five categories (1) continuation of triggering NSAID order, (2) dose modification, (3) alternative NSAID ordered without PGx implications, (4) alternative analgesic (ie, opioid) ordered, and (5) discontinuation of NSAID without alternative therapy.

**Results:**

The study analyzed 2361 alert instances from 978 patients. The most common response was discontinuing NSAID without alternative therapy (43%). Dose modifications and orders for alternative analgesics comprised 2.57% and 14.67% of responses, respectively. The initial acceptance rate was 62.6%. Prior NSAID use significantly impacted override rates (60% vs 40%, *P* < .001). A 409-day breaking point was observed to affect alert acceptance rates, with the highest acceptance in NSAID naïve patients (96.1%).

**Discussion:**

PGx NSAIDs CDS alert acceptance rates were higher compared to general CDS acceptance rates. This study highlights opportunities for continuous improvement including optimizing alert modality, modifying alert criteria to include look-back periods, and implementing genetically adapted ordersets.

**Conclusion:**

The initial acceptance rate of PGx NSAIDs CDS alerts was 62.6%, however, with significantly higher acceptance rates in NSAID naïve patients (62.6% vs 96.1%, *P* < .001). Integration of CDS is vital to the successful implementation of PGx in clinical practice.

## Background and significance

Genetics can influence medication response. This field is called pharmacogenomics (PGx). Evidence based PGx guidance is available for several medications to aid clinicians in medication selection to improve efficacy and/or decrease risk of adverse effects.^1^ However, there are many barriers and limitations utilizing PGx within clinical practice. One of the largest barriers of PGx implementation is the incorporation into an already busy clinician workflow to increase proficiency of using PGx recommendations.^2–4^ One strategy is to incorporate PGx recommendations into clinical decision support (CDS) alerts. Two common alert modalities include interruptive alerts (pop up alerts) which require acknowledgement prior to proceeding within the workflow and non-interruptive alerts (in-line alerts) which provide information without a forced stop in the workflow.^5^^,^^6^ Although alerts can potentially improve prescribing practices, the alerts are only as effective as their design and content. Alerts that require checking of a box or response can draw attention to urgent patient-specific factors but can be overly disruptive and be a barrier to the clinician engaging with the content displayed in addition to contributing to alert fatigue.^2^

Nonsteroidal anti-inflammatory drugs (NSAIDs) are a commonly prescribed class of medications in which PGx guidance is available to help minimize adverse effects. Although NSAIDs may be considered generally well tolerated and benign medications, with long-term usage at high doses they can lead to serious adverse effects such as increased risk of developing an upper gastrointestinal bleed.^7^  *CYP2C9* is responsible for the metabolism of several NSAIDs. Variants in this gene can lead to patients presenting as CYP2C9 intermediate or poor metabolizers. Decreased metabolism can lead to an accumulation of NSAID serum concentrations that may increase the risk of adverse side effects for these patients.^7^ The Clinical Pharmacogenetics Implementation Consortium (CPIC) guidelines currently provide dosing recommendations for celecoxib, flurbiprofen, lornoxicam, ibuprofen, meloxicam, piroxicam, and tenoxicam.^8^ Many health systems have developed PGx CDS to guide NSAID prescribing, however, the effectiveness of PGx alerts has not been well documented in the literature.^9–12^

## Objective

The objective of this study was to evaluate clinician response rates to PGx NSAIDs CDS alerts and identify factors which may impact adherence to alert recommendations.

## Materials and methods

**Figure 1.**
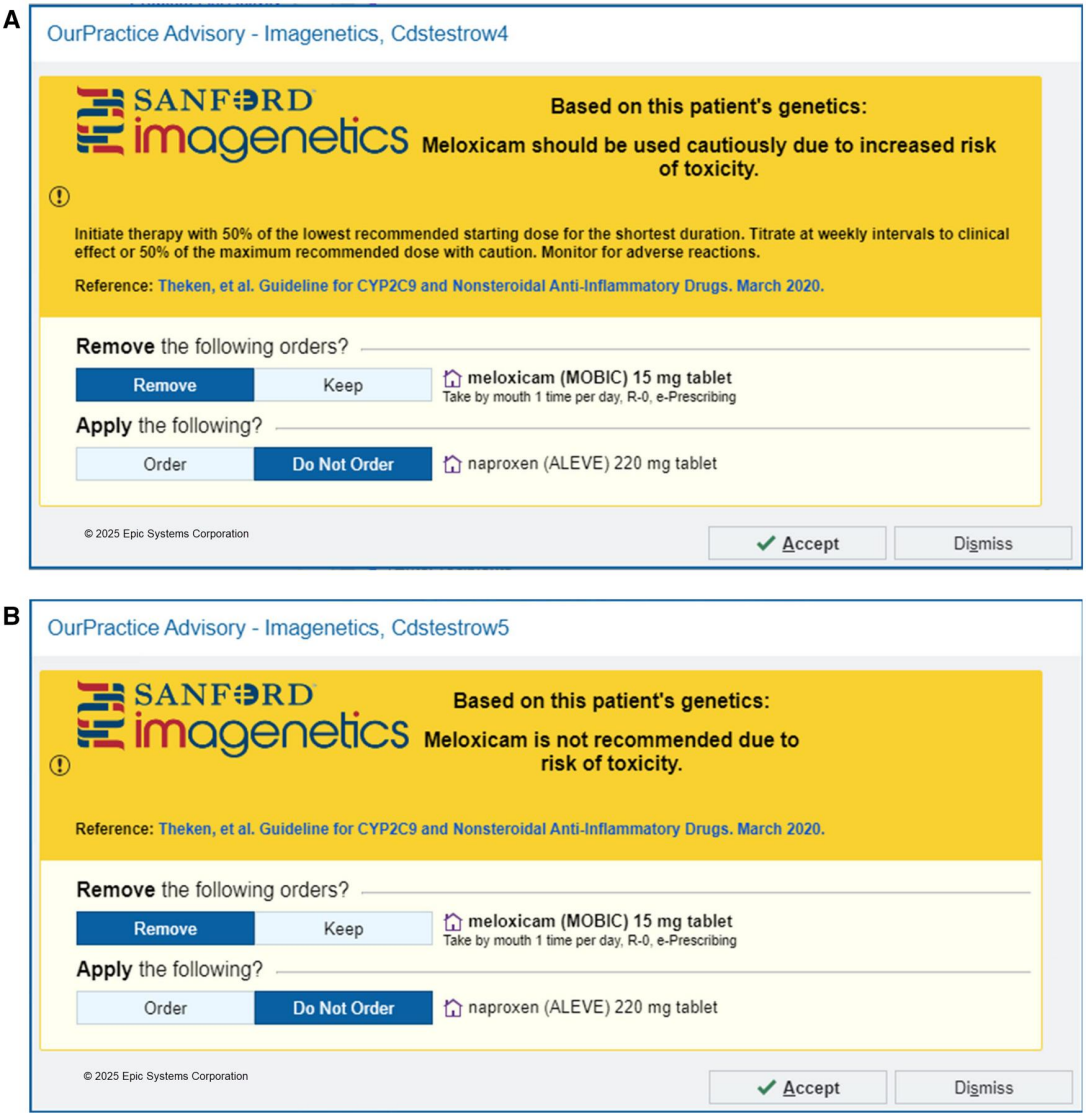
Example of interruptive Nonsteroidal Anti-Inflammatory Drugs (NSAIDs) Clinical Decision Support (CDS) Alerts for CYP2C9 Intermediate Metabolizers (A) and CYP2C9 Poor Metabolizers (B).

CDS, primarily through interruptive alerts, has been the mainstay of prospective notification for drug-gene interactions at Sanford Health.^13^ Post test interruptive alerts were implemented in May 2020 for PGx guided NSAIDs (ibuprofen, celecoxib, flurbiprofen, meloxicam and piroxicam as these products are readily available in the United States) in alignment with CPIC guidelines.^8^ Each of the five CDS alerts share similar logic surrounding diplotypes based on activity scores (intermediate metabolizers with activity score of 1 and poor metabolizers with activity score of 0-0.5) and corresponding medication criteria. CDS alert rules can be found within the Supplementary Materials. These alerts describe the clinical impact of the drug-gene interaction, default to remove the offending NSAID, and offer a structured response option to order naproxen which would be a genetically appropriate NSAID (Figure 1). The PGx NSAIDs CDS alerts trigger at multiple steps in the medication use process (order entry and order signing) on new and refill prescriptions. In April of 2023, NSAID alerts were suppressed from triggering within surgical areas due to the short duration of the active order within the phase of care in an effort to minimize alert fatigue. A retrospective chart review was conducted at Sanford Health to analyze response rates of PGx NSAIDs CDS alerts. This study was approved by the Sanford Health Institutional Review Board STUDY00003518.

PGx NSAIDs interruptive alerts were abstracted from the electronic medical record (EMR) and analyzed from May 2020 through December 2024. The primary endpoint of interest was how clinicians responded to an alert. Specifically, clinicians could have responded in one or more of five different ways: (1) continuation of an NSAID order, (2) dose modification, (3) place an order for an alternative NSAID without PGx implications, (4) place an order for an alternative analgesic (ie, opioid), or (5) removal of an NSAID order in which no alternative therapy was ordered. For example, a clinician may elect for a dose reduction of the offending NSAID and prescribe an alternative analgesic, therefore, categorized into two distinct response options. The presence of orders being connected to an orderset (group of orders related to a specific indication), whether the responding order was a refill, time from last refill, administration route, department specialty, patient demographics (eg, age, sex, race), and *CYP2C9* corresponding activity score (when available) and phenotype were considered as potential contextual variables that could influence responding orders.

Descriptive frequency statistics were evaluated to understand the rate of alert acceptance and how that acceptance rate compares under different contexts. Namely, this context was theorized to differ largely depending on whether the patient had a previous NSAID order and whether the responding order was connected to an orderset. A final dichotomized response to alert orders was categorized as a label for more controlled multivariate analysis. Specifically, if an order for a PGx guided NSAID was placed despite the alert, then it was labeled as *overridden*. Accepted response options were defined as a dose modification, an order for an alternative NSAID (non-PGx guided), an order for an alternative analgesic, or removal of a triggering NSAID order. Initial statistical significance with an *accepted* vs *overridden* response was evaluated using Wilcoxon rank sum tests for numeric covariates and chi squared and Fisher’s exact tests for nominal covariates.

A multiple logistic regression model was fit whereby covariates that reveal statistical significance were inputted in a reverse stepwise method to predict whether an alert was *accepted* vs *overridden*. Given that patients could be exposed to multiple alerts, the unique patient identifier (ID) was treated as a random effect.

## Results

A total of 978 patients comprised 2361 alert instances. A total of 2842 clinician responses were derived from the 2361 alert instances. All analyses were completed utilizing the total number of alert instances. Meloxicam was the most common NSAID to trigger PGx NSAIDs CDS alerts (47.8%) followed by ibuprofen (30.6%) and celecoxib (21.3%). Flurbiprofen and piroxicam comprised 0.21% and 0.12% respectively. The two most common responses to an alert were removal of NSAID without an *additional order* and *continuation of the NSAID order*, 43% and 33.88% respectively (Table 1).

**Table 1.** Responses to Pharmacogenomics (PGx) Clinical Decision Support (CDS) Alerts for Nonsteroidal Anti-Inflammatory Drugs (NSAIDs) stratified by triggering orderset utilization and prescription history.

	Alternative NSAID	Alternative analgesic (ie, Opioid)	Continuation of NSAID	Dose modification	Removal of NSAID
Orderset status^a^					
From orderset (*N* = 487)	68 (14%)	208 (43%)	133 (27%)	13 (3%)	65 (13%)
No orderset (*N* = 2355)	99 (4%)	209 (9%)	830 (35%)	60 (3%)	1157 (49%)
Overall	167 (5.88%)	417 (14.67%)	963 (33.88%)	73 (2.57%)	1222 (43%)
NSAID history^a^					
First NSAID (*N* = 1047)	40 (3.82%)	108 (10.32%)	43 (4.11%)	6 (0.57%)	850 (81.18%)
Had Prior NSAID (*N =* 1795)	127 (7.08%)	309 (17.21%)	920 (51.25%)	67 (3.73%)	372 (20.72%)

a Alert instances = 2361 with a total of 2842 response options as clinicians may respond to the alert with multiple options for each alert instance.

An order for an alternative analgesic comprised 14.67% of all alert responses followed by ordering alternative NSAIDs (5.88%). Naproxen accounted for a majority of alternative responding NSAID orders (55.26%). Dose modifications accounted for 2.57% of responses to alerts. Only alerts which triggered based celecoxib, meloxicam, and ibuprofen resulted in dose modifications (Table 2). Alerts for flurbiprofen and piroxicam did not yield any dose adjustments. CYP2C9 intermediate metabolizers accounted for 86% (63 of the 73) dose modifications. A total of 7 dose modifications responses were attributed to CYP2C9 poor metabolizers for either celecoxib or ibuprofen. Whereas CYP2C9 poor metabolizers and orders for meloxicam accounted for 3 alert responses resulting in dose modification. According to our method of categorizing any alert-related NSAID order without a dose change as overridden, the initial acceptance rate was observed as 62.6%.

**Table 2.** Responses to Pharmacogenomics (PGx) Clinical Decision Support (CDS) Alerts for Nonsteroidal Anti-Inflammatory Drugs (NSAIDs) stratified by medication and CYP2C9 phenotype.

	Alternative NSAID	Continuation of NSAID	Dose modification	Removal of NSAID	Alternative analgesic (ie, Opioid)
Celecoxib					
Intermediate Metabolizer	35 (6.49%)	290 (53.8%)	26 (4.82%)	112 (20.78%)	76 (14.1%)
Poor Metabolizer	6 (6.19%)	40 (41.24%)	4 (4.12%)	26 (26.8%)	21 (21.65%)
Flurbiprofen					
Intermediate Metabolizer	0 (0%)	1 (20%)	0 (0%)	3 (60%)	1 (20%)
Ibuprofen					
Intermediate Metabolizer	83 (9.78%)	157 (18.49%)	18 (2.12%)	362 (42.64%)	229 (26.97%)
Poor Metabolizer	10 (6.94%)	29 (20.14%)	3 (2.08%)	69 (47.92%)	33 (22.92%)
Meloxicam					
Intermediate Metabolizer	26 (2.49%)	401 (38.41%)	19 (1.82%)	546 (52.3%)	52 (4.98%)
Poor Metabolizer	7 (4.35%)	45 (27.95%)	3 (1.86%)	101 (62.73%)	5 (3.11%)
Piroxicam					
Intermediate Metabolizer	0 (0%)	0 (0%)	0 (0%)	3 (100%)	0 (0%)

**Figure 2.**
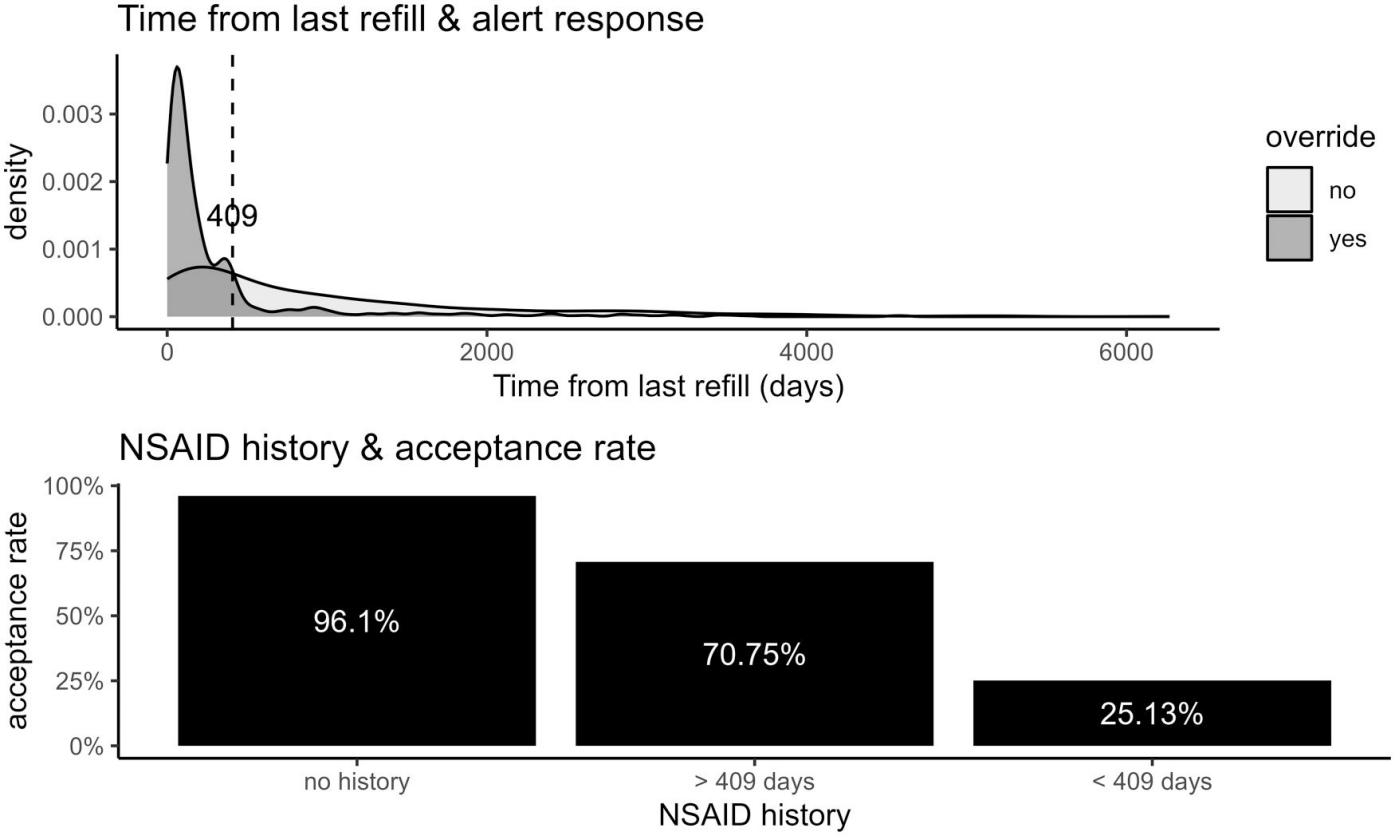
Nonsteroidal Anti-Inflammatory Drugs (NSAIDs) prescription history & Clinical Decision Support (CDS) Alerts Responses.

Alerts generated from NSAID orders placed from ordersets comprised 11% of all alert instances. Opioid prescriptions were higher in the analysis examining the use of ordersets in response to the alert, 43% as opposed to 9% when an orderset was not utilized (Table 1). Overridden alerts, the continuation of a PGx-guided NSAID despite the alert, was largely impacted by the presence of previous prescription history for NSAIDs. Clinicians overrode the alert 51.25% of the time when the patient had a previous prescription for PGx-guided NSAIDs compared to 4.11% of the time for individuals who were NSAID naïve (Table 1). Furthermore, we found that the probability of an overridden response is much higher proximally to the last refill as opposed to when more time has elapsed (*r* = 0.42, *P* < .001). The time between the last NSAID order impacting the alert response was approximately 409 days determined by calculating the number of days from the most recent NSAID order and the date of the PGx NSAIDs CDS alert triggering and examining the point of separation between those alert acceptance vs overridden (Figure 2). When categorized to this breaking point of 409 days, the observed alert acceptance was the highest in NSAID naïve patients (96.1%), followed by alerts which had previous NSAID prescriptions beyond 409 days (70.8%), and the lowest alert acceptance when the alert generated was less than 409 days from the last NSAID prescription (25.1%). All alert responses were labeled as *overridden* vs *accepted* for a solidified multivariate model, where the goal was to attempt to determine an overridden response. All baseline alert characteristics can be seen in Table 3.

**Table 3.** Overridden multivariate analysis baseline characteristics.

	Alert Instances *N* = 2361		
Characteristic	**Accepted** *N* = 1479^a^	**Overridden** *N* = 882^a^	***P*-value** ^b^
**Year**			.3
*2020*	140 (60%)	92 (40%)	
*2021*	265 (64%)	148 (36%)	
*2022*	339 (66%)	174 (34%)	
*2023*	366 (62%)	225 (38%)	
*2024*	369 (60%)	243 (40%)	
**Alerts Instances**	2.01 (1.3)	2.23 (1.5)	<.001^***^
**Age**	55 (40, 66)	56 (46, 67)	.002^**^
**Sex**			.10
*Female*	1106 (64%)	632 (36%)	
*Male*	373 (60%)	250 (40%)	
**Race**			.2
*African American/Black*	2 (100%)	0 (0%)	
*American Indian or Alaskan Native*	24 (57%)	18 (43%)	
*Asian*	7 (100%)	0 (0%)	
*Caucasian/White*	1441 (63%)	861 (37%)	
*Declined*	5 (63%)	3 (38%)	
**CYP2C9 Metabolizer Status**			.005^**^
*Intermediate Metabolizer*	1241(61%)	778 (39%)	
*Poor Metabolizer*	238 (70%)	104 (30%)	
**Specialty**			<.001^***^
*Acute Care*	26 (90%)	3 (10%)	
*Emergency*	72 (96%)	3 (4.0%)	
*Family/Internal Medicine*	485 (48%)	523 (52%)	
*General Surgery*	29 (83%)	6 (17%)	
*Inpatient*	396 (86%)	66 (14%)	
*OBGYN*	92 (59%)	64 (41%)	
*Ortho*	255 (69%)	116 (31%)	
*Pain*	30 (56%)	24 (44%)	
*Rheumatology*	48 (43%)	64 (57%)	
*Other*	46 (78%)	13 (22%)	
**From Orderset**	148 (57%)	112 (43%)	.043^*^
**Prior NSAID**	567 (40%)	845 (60%)	<.001^***^

a n (%); Mean (SD); Median (IQR).

b *P < .05; **P < .01; ***P < .001.

Patients that were slightly older had a higher override rate (median = 56 vs 55 respectively) (*P* < .01). The sample was largely female, with a non-significant trend that they had a slightly lower override rate (36% vs 40%, *P* = .1). The number of alerts for a given individual increased the likelihood that an alert was overridden (mean = 2.23) vs being accepted (mean = 2.01) (*P* < .001). Alerts that generated for CYP2C9 poor metabolizers had a lower override rate compared to alerts for CYP2C9 intermediate metabolizers, 30% compared to 39% respectively (*P* < .01).

The department specialty had a differential effect on the override rate (*P* < .001). Primary care (family medicine/internal medicine) had a very large override rate (52%), especially given that it represented a substantial percentage of all alerts (43%). The presence of an orderset increased the likelihood of an overridden alert (57% vs 43%, *P* < .05). Prior NSAID use had a large effect on an alert being overridden (60% vs 40%, *P* < .001).

A mixed effects logistic regression model was fit and evaluated whereby alert overrides were regressed on previous statistically justified covariates with patient ID as random effect (Table 4).

**Table 4.** Overridden response multivariate model coefficients.

Characteristic		OR^a^	**SE** ^a^	**95% CI** ^a^	*P*-value
Poor metabolizer		0.67	0.149	0.43, 1.03	.07
Primary care departments		2.72	0.421	2.00, 3.68	<.001
From orderset		2.7	0.59	1.76, 4.14	<.001
Prior NSAID		39.3	8.04	26.3, 58.6	<.001

a OR = Odds Ratio, SE = Standard Error, CI = Confidence Interval.

Alerts for CYP2C9 poor metabolizers trended toward a decrease in the likelihood of an override in comparison to alerts for intermediate metabolizers (OR = 0.67 [0.43, 1.03], *P* = .07). Primary care departments had a greater likelihood of overrides compared to all other specialties (OR = 2.72 [2.00, 3.68], *P* < .001). The presence of an orderset increased the likelihood of an override (OR = 2.7 [1.76, 4.14], *P* < .001). Prior NSAID use greatly increased the likelihood of an override (OR = 39.3 [26.3, 58.6], *P* < .001). Patient age and number of alert instances did not prove to be statistically significant in the presence of other covariates (*P* > .05). The intraclass correlation coefficient (ICC) was revealed to be 0.248. The repeated measure effect accounted for 61.1% of the conditional variance while all fixed effects explained 48.3% of the marginal variance.

## Discussion

Traditionally, PGx CDS acceptance has been categorized as accepted or overridden based on structured responses within the alert itself.^10^^,^^14–16^ This study highlights additional metrics such as dose modifications that can be utilized to further define the success of PGx alert implementation. The CPIC guidelines for NSAIDs recommend alternative dosing in lieu of transitioning to alternative therapies based on CYP2C9 activity score when clinically appropriate.^8^ Ninety-six percent of dose modifications were in alignment with CPIC guidelines. Only 4% of dose modifications were made despite alert recommendations to avoid meloxicam in CYP2C9 poor metabolizers. Historically, alternative dosing in response to PGx CDS has been difficult to capture as in many cases it has required manual data collection.^15^^,^^17^^,^^18^ Additionally, the incorporation of order origin (ie, orderset derived NSAID orders and subsequent responses with alternative orders) can be utilized to further investigate clinician response.

The overall alert acceptance rate for PGx NSAIDs CDS was 63%, however, increased significantly in those who were NSAID naïve (96.1%). Published literature suggests that PGx CDS acceptance rates are highly variable depending on the severity of the drug-gene interaction with acceptance rates ranging from 19.5% to 92%.^10^^,^^14–19^ Thus establishing ideal targets for PGx CDS benchmarking is difficult. Nguyen and colleagues reported 75% adherence to CDS alert recommendations on a small subset (*N* = 16) of alerts in a pediatric hospital which included ibuprofen, meloxicam, amitriptyline, imipramine, atazanavir, fluoxetine, and tacrolimus.^10^ While not specific to PGx CDS, literature suggests a wide range of alert override rates from 49%-96% of alerts.^20^

The most common response to the NSAID alerts resulted in the removal of the offending NSAID without alternative therapy (43%). This percentage drastically increases (81%) when evaluating the first NSAID prescription. The prescriber may have reconsidered the need for an analgesic based on the alert. Prescribing an opioid (alternative analgesic) may be associated with additional risks compared to NSAIDs, however, a multimodal approach to pain management may be necessary based on pain severity. Selection of an opioid could also include those patients that would have received an opioid anyway with an additional prescription for an NSAID.

The CPIC guidelines have differing recommendations depending on the specific NSAID, CYP2C9 activity score, and phenotype.^8^ Generally, for CYP2C9 intermediate metabolizers, the guideline recommends dose modification whereas poor metabolizers should avoid use and select alternative therapy. CYP2C9 poor metabolizers are more likely to experience side effects and toxicities due to increased plasma concentrations. The language within the PGx NSAIDs CDS alerts mirrors CPIC guideline recommendations (Figure 1A and B). Metabolizer status is an important consideration when evaluating alert response as it might provide insights into prescribing based on increased risk and severity. These reasons could contribute to the lower override rate for CYP2C9 poor metabolizers. Dose modifications accounted for 2.57% of NSAID alert response. While this is a relatively low number, the ability to discretely capture dose modifications without manual review and data collection may provide additional insights to alert optimizations. Previous work evaluating the effect of interruptive vs non-interruptive alerts suggested that while interruptive alerts were accepted more frequently through selecting a new medication, non-interruptive alerts were more effective for dose changes.^18^ This provides another consideration when designing alerts and suggests non-interruptive alerts for CYP2C9 intermediate metabolizers may facilitate a dose decrease more than an interruptive alert. Therefore, the incorporation of dose criteria may reduce the volume of alerts and enhance the sensitivity of CDS alerts.^21^

The difference shown between opioid orders generated from ordersets vs not indicates that opioid orders were not a large percentage of what might be deemed *alternatives* to the alert, but more so, as part of analgesic medication grouping within an orderset. In other words, regardless of genetics, an opioid prescription would have been likely. NSAIDs were predominately discontinued when standard order entry was utilized as opposed to orders generated from ordersets. Given the limited amount of time NSAIDs orders were discontinued when ordersets were utilized, suggests additional systemic approaches should be considered such as genetically adaptable ordersets. The utilization of genetically adapted ordersets can suppress certain medications with drug-gene interactions from displaying when genetic variants are identified for a patient.^22^ The ability to filter out orderset medications based on PGx could be impactful in across multiple therapeutic options for pain (not only for *CYP2C9* and NSAIDS but also for *CYP2D6* and opioids). Dose modifications were infrequent in NSAID orders stemming from ordersets. Therefore, another potential enhancement within ordersets is to add criteria for dose optimization directly within the orderset to only display genetically appropriate doses.

An increase in the likelihood an alert would be overridden was seen for each additional alert triggered. One explanation could be that our alerts generated for both new prescriptions and refills. Prior use of the triggering NSAID largely impacted if the alert was overridden. If the patient is tolerating the medication well currently or had the same prescription in the past that was well tolerated, the clinician may be less likely to accept the alert and prescribe an alternative therapy. An area worthy of future investigation is the repetitive alerts despite clinicians’ conscious decision to continue with an NSAID order. As alert functionality continues to evolve, it might be worthwhile to include dose matching criteria to automatically suppress alerts from triggering if a patient previously tolerated an NSAID order.

Given that the alert is more likely to be accepted the longer the duration between prescriptions, it may be reasonable to consider modifying alert triggering criteria, especially for intermediate metabolizers. With a breaking point of 409 days as a cutoff for higher alert acceptance, perhaps the ability to “snooze” the alert for a year could decrease alert frequency and potential alert fatigue. Another approach could be to suppress the alert until after a certain number of days (ie, 409 or, more practically 365 days) by utilizing look back features to minimize alert instances. Alternatively, transitioning from interruptive to a non-interruptive alert may be beneficial.

While not specific to PGx, Gill and colleagues conducted a randomized controlled trial evaluating the role of CDS and adherence to guidelines for patients taking NSAIDs.^23^ CDS assisted in a modest improvement in guideline-concordant care within the intervention arm compared to control arm. While this study showed a statistical significance in guideline-concordance through use of CDS, it also highlighted the difficulties in designing CDS in a meaningful way, impactful for patient care. Designing and refining alerts to fit into clinical situations and workflows are necessary to improve patient care. Inclusion of parameters such as patient age and serum creatinine in addition to PGx results creates multifactorial CDS logic to further mitigate risk for adverse effects and minimize the potential for alert fatigue given our currently broad alert criteria.

This study is not without limitations. CYP2C9 activity score has not always been included in the return of results. Some data did not have CYP2C9 activity score for interpretation, however, diplotype values were utilized with CDS logic to ensure alerts only presented for impacted patients. Select NSAIDs are widely available as over the counter medications, therefore, may not generate CDS alerts if not properly documented within the EMR. Alerts only fire upon prescription actions (order entry and signing) and not reconciliation of medications. Many comorbid conditions could affect the appropriateness of NSAID prescribing and these conditions were not evaluated within this study. Retrospective evaluations inherently yield some limitations as all factors may not be controlled for during analysis. These results may not be generalizable to all healthcare settings as our analysis was conducted at a single healthcare institution in which clinicians received mandatory PGx education.^24^ Multiple studies examine NSAID efficacy and/or safety/toxicities in the context of PGx.^25^ Clinical outcomes were not collected as part of this analysis. However, this study is impactful as it is the first study to our knowledge to evaluate response to PGx NSAIDs CDS alerts on a large scale.

## Conclusion

Integration of CDS is vital to the successful implementation of PGx in clinical practice. This study conducted a thorough analysis of clinician responses to PGx NSAIDs CDS alerts. Although the findings showed a baseline acceptance rate of nearly 63%, when focusing on NSAID naïve patients the acceptance rate was much higher (96.1%). Dose modifications represented a small portion of accepted responses; however, this has historically required manual chart review and could be underrepresented in the literature. Many avenues exist to provide PGx guidance, nonetheless, continuous monitoring of CDS is needed to optimize CDS modalities and prevent alert fatigue. Future directions to optimize PGx NSAIDs alerts within our organization will be a multilayered approach including transitioning to non-interruptive alerts with inclusion of dose criteria and development of genetically adaptable ordersets. Future research to incorporate clinical outcomes is warranted to ascertain the effects of PGx-guided prescribing.

## Supplementary Material

ooaf112_Supplementary_Data

## Data Availability

The data underlying this article cannot be shared publicly due to privacy concerns. Researchers interested in data access and collaboration are encouraged to contact the corresponding author.
