# Supplementary material for: Analyzing the impact of pharmacogenomics-guided nonsteroidal anti-inflammatory drug alerts in clinical practice
Source: JAMIA Open. 2025 Oct 3;8(5):ooaf112. doi: 10.1093/jamiaopen/ooaf112 (PMC12492481; doi:10.1093/jamiaopen/ooaf112)
Supplement: ooaf112_Supplementary_Data [file ooaf112_supplementary_data.docx]

# SUPPLEMENTAL MATERIALS

**Clinical decision support (CDS) alert rules for meloxicam-CYP2C9 with activity score of 1 (intermediate metabolizers):**

**Display:** [Sanford Imagenetics logo] Based on this patient's genetics: Meloxicam should be used cautiously due to increased risk of toxicity.

Initiate therapy with 50% of the lowest recommended starting dose for the shortest duration. Titrate at weekly intervals to clinical effect or 50% of the maximum recommended dose with caution.  Monitor for adverse reactions

Hyperlink to CPIC guidelines: Theken, et al. Guideline for CYP2C9 and Nonsteroidal Anti-Inflammatory Drugs. March 2020.

**Criteria:**

- Meloxicam via simple generic (6610005200, 6610005260, 6699800250)

AND

- CYP2C9 diplotypes with activity score 1 (intermediate metabolizers)

| *1/*3 | *1/*6 | *1/*13 | *1/*15 | *2/*2 |
| --- | --- | --- | --- | --- |
| *2/*4 | *2/*5 | *2/*8 | *2/*11 | *2/*12 |
| *2/*16 | *4/*4 | *4/*5 | *4/*8 | *4/*11 |
| *4/*12 | *4/*16 | *5/*5 | *5/*8 | *5/*11 |
| *5/*12 | *5/*16 | *8/*8 | *8/*11 | *8/*12 |
| *8/*16 | *11/*11 | *11/*12 | *11/*16 | *12/*12 |
| *12/*16 | *16/*16 |  |  |  |

**Restrictions:**

- Exclude intraoperative

**Triggers:**

- Enter orders
- Sign orders
- Select item in Order Set, SmartSet or Pathway

**Actions:**

- Suggest removal of all triggering unsigned orders
- Ability to order naproxen

**Clinical decision support (CDS) alert rules for meloxicam-CYP2C9 poor metabolizers:**

**Display:** [Sanford Imagenetics logo]Based on this patient's genetics: Meloxicam is not recommended due to risk of toxicity.

Hyperlink to CPIC guidelines: Theken, et al. Guideline for CYP2C9 and Nonsteroidal Anti-Inflammatory Drugs. March 2020.

**Criteria:**

- Meloxicam via simple generic (6610005200, 6610005260, 6699800250)

AND

- CYP2C9 diplotypes with activity score 0-0.5 (poor metabolizers)

| *2/*3 | *2/*6 | *2/*13 | *2/*15 | *3/*3 |
| --- | --- | --- | --- | --- |
| *3/*4 | *3/*5 | *3/*6 | *3/*8 | *3/*11 |
| *3/*12 | *3/*13 | *3/*15 | *3/*16 | *4/*6 |
| *4/*13 | *4/*15 | *5/*6 | *5/*13 | *5/*15 |
| *6/*6 | *6/*8 | *6/*11 | *6/*12 | *6/*13 |
| *6/*15 | *6/*16 | *8/*13 | *8/*15 | *11/*13 |
| *11/*15 | *12/*13 | *12/*15 | *13/*13 | *13/*15 |
| *13/*16 | *15/*15 | *15/*16 |  |  |

**Restrictions:**

- Exclude intraoperative

**Triggers:**

- Enter orders
- Sign orders
- Select item in Order Set, SmartSet or Pathway

**Actions:**

- Suggest removal of all triggering unsigned orders
- Ability to order naproxen

**Clinical decision support (CDS) alert rules for piroxicam-CYP2C9 activity score 0-1 (intermediate and poor metabolizers):**

**Display:** [Sanford Imagenetics logo] Based on this patient's genetics: Piroxicam is not recommended due to risk of toxicity.

Hyperlink to CPIC guidelines: Theken, et al. Guideline for CYP2C9 and Nonsteroidal Anti-Inflammatory Drugs. March 2020.

**Criteria:**

- Piroxicam via simple generic (6610007000, 6699800270) **AND**
- CYP2C9 diplotypes with activity score 1 (intermediate metabolizers) **OR**

| *1/*3 | *1/*6 | *1/*13 | *1/*15 | *2/*2 |
| --- | --- | --- | --- | --- |
| *2/*4 | *2/*5 | *2/*8 | *2/*11 | *2/*12 |
| *2/*16 | *4/*4 | *4/*5 | *4/*8 | *4/*11 |
| *4/*12 | *4/*16 | *5/*5 | *5/*8 | *5/*11 |
| *5/*12 | *5/*16 | *8/*8 | *8/*11 | *8/*12 |
| *8/*16 | *11/*11 | *11/*12 | *11/*16 | *12/*12 |
| *12/*16 | *16/*16 |  |  |  |

- CYP2C9 diplotypes with activity score 0-0.5 (poor metabolizers)

| *2/*3 | *2/*6 | *2/*13 | *2/*15 | *3/*3 |
| --- | --- | --- | --- | --- |
| *3/*4 | *3/*5 | *3/*6 | *3/*8 | *3/*11 |
| *3/*12 | *3/*13 | *3/*15 | *3/*16 | *4/*6 |
| *4/*13 | *4/*15 | *5/*6 | *5/*13 | *5/*15 |
| *6/*6 | *6/*8 | *6/*11 | *6/*12 | *6/*13 |
| *6/*15 | *6/*16 | *8/*13 | *8/*15 | *11/*13 |
| *11/*15 | *12/*13 | *12/*15 | *13/*13 | *13/*15 |
| *13/*16 | *15/*15 | *15/*16 |  |  |

**Restrictions:**

- Exclude intraoperative

**Triggers:**

- Enter orders
- Sign orders
- Select item in Order Set, SmartSet or Pathway

**Actions:**

- Suggest removal of all triggering unsigned orders
- Ability to order naproxen

**Clinical decision support (CDS) alert rules for ibuprofen, flurbiprofen, or celecoxib-CYP2C9 with activity score of 1 (intermediate metabolizers):**

**Display:** [Sanford Imagenetics logo] Based on this patient's genetics: Celecoxib, flurbiprofen, and ibuprofen should be used cautiously due to increased risk of toxicity.

Initiate with the lowest possible starting dose for the shortest duration.  Titrate dose to clinical response cautiously.  Monitor for adverse reactions.

Hyperlink to CPIC guidelines: Theken, et al. Guideline for CYP2C9 and Nonsteroidal Anti-Inflammatory Drugs. March 2020.

**Criteria:**

- Celecoxib via simple generic (6610052500, 3499870210, 6760403000, 6599500210) **OR**
- Flurbiprofen via simple generic (6610001200) **OR**
- Ibuprofen via simple generic (4399100231, 4399100240, 4399400311, 4399400377, 6030990240, 6030990242, 6599000226, 6599170250, 6610002000, 4399100231, 6599000226, 6610002040, 6610002050, 6610990230, 6610990232, 6610990328, 6699100440, 6699800240, 6699800275, 6699850230)

**AND**

- CYP2C9 diplotypes with activity score 1 (intermediate metabolizers)

| *1/*3 | *1/*6 | *1/*13 | *1/*15 | *2/*2 |
| --- | --- | --- | --- | --- |
| *2/*4 | *2/*5 | *2/*8 | *2/*11 | *2/*12 |
| *2/*16 | *4/*4 | *4/*5 | *4/*8 | *4/*11 |
| *4/*12 | *4/*16 | *5/*5 | *5/*8 | *5/*11 |
| *5/*12 | *5/*16 | *8/*8 | *8/*11 | *8/*12 |
| *8/*16 | *11/*11 | *11/*12 | *11/*16 | *12/*12 |
| *12/*16 | *16/*16 |  |  |  |

**Restrictions:**

- Exclude intraoperative

**Triggers:**

- Enter orders
- Sign orders
- Select item in Order Set, SmartSet or Pathway

**Actions:**

- Suggest removal of all triggering unsigned orders
- Ability to order naproxen

**Clinical decision support (CDS) alert rules for ibuprofen, flurbiprofen, or celecoxib-CYP2C9 poor metabolizers:**

**Display:** [Sanford Imagenetics logo] Based on this patient's genetics: Celecoxib, flurbiprofen, and ibuprofen should be used cautiously due to increased risk of toxicity.

Initiate therapy with 25-50% of the lowest recommended starting dose for the shortest duration.  Titrate at weekly intervals to clinical effect or 25-50% of the maximum recommended dose.  Monitor for adverse reactions.

Hyperlink to CPIC guidelines: Theken, et al. Guideline for CYP2C9 and Nonsteroidal Anti-Inflammatory Drugs. March 2020.

**Criteria:**

- Celecoxib via simple generic (6610052500, 3499870210, 6760403000, 6599500210) **OR**
- Flurbiprofen via simple generic (6610001200) **OR**
- Ibuprofen via simple generic (4399100231, 4399100240, 4399400311, 4399400377, 6030990240, 6030990242, 6599000226, 6599170250, 6610002000, 4399100231, 6599000226, 6610002040, 6610002050, 6610990230, 6610990232, 6610990328, 6699100440, 6699800240, 6699800275, 6699850230)

**AND**

- CYP2C9 diplotypes with activity score 0-0.5 (poor metabolizers)

| *2/*3 | *2/*6 | *2/*13 | *2/*15 | *3/*3 |
| --- | --- | --- | --- | --- |
| *3/*4 | *3/*5 | *3/*6 | *3/*8 | *3/*11 |
| *3/*12 | *3/*13 | *3/*15 | *3/*16 | *4/*6 |
| *4/*13 | *4/*15 | *5/*6 | *5/*13 | *5/*15 |
| *6/*6 | *6/*8 | *6/*11 | *6/*12 | *6/*13 |
| *6/*15 | *6/*16 | *8/*13 | *8/*15 | *11/*13 |
| *11/*15 | *12/*13 | *12/*15 | *13/*13 | *13/*15 |
| *13/*16 | *15/*15 | *15/*16 |  |  |

**Restrictions:**

- Exclude intraoperative

**Triggers:**

- Enter orders
- Sign orders
- Select item in Order Set, SmartSet or Pathway

**Actions:**

- Suggest removal of all triggering unsigned orders
- Ability to order naproxen
